# Enhancing resilience to landslide disaster risks through rehabilitation of slide scars by local communities in Mt Elgon, Uganda

**DOI:** 10.4102/jamba.v9i1.390

**Published:** 2017-05-31

**Authors:** Bob R. Nakileza, Mwajalolo J. Majaliwa, Abu Wandera, Clare M. Nantumbwe

**Affiliations:** 1 Department of Environment Management, Makerere University, Uganda; 2 Department of Geography, Geo Informatics and Climatic Sciences, Makerere University, Uganda; 3 Small Grants Programme/United Nations Programme, Uganda

## Abstract

Mass movements are key drivers affecting the utilisation of many farmlands and consequently the livelihoods in mountains’ ecosystems. Numerous expansive landslide scars can for years remain unusable for crop farming purposes, which is a major livelihood activity. This article examined the approaches and challenges faced by local communities in the rehabilitation of landslide-degraded areas in selected areas of Mt Elgon. Data were collected through field surveys of purposively selected scars, key informant interviews and focus group discussions with the local communities. The findings indicate that the local communities have initiated the rehabilitation of some scars to stabilise the slopes and also accelerate their quick recovery for beneficial purposes. Community trainings coupled with awareness and participatory actions during rehabilitation enhance community preparedness to landslide risks. However, there were noted constraints including limited resources, incidences of secondary slides, cracks and lack of adequate knowledge on the existing best practices for the rehabilitation of scars on deeply weathered soils. Further research should be focussed on generating relevant knowledge on regeneration rates under different socio-ecological conditions and for guiding sustainable utilisation of fragile areas.

## Introduction

A landslide is a downslope gravitational movement of a mass of earth or rock as a unit owing to failure of the material (Highland & Bobrowsky 2008). Landslides are a neutral phenomenon and a normal feature of landscapes experiencing dissections, but their magnitude, frequency and geographical distribution have been considerably modified in recent years by human intervention (Jones 1992). Human activities, such as poor logging practices and over-cultivation on unstable steep slopes, accelerate landslide occurrence, but activities including deforestation, farm cultivation, forestry restoration and prevention work are the key factors influencing the timing of landslide density changes in the short term (Chang & Slaymaker 2002). Landslides and mass movements are historical problems in the highlands of East Africa (Bagoora 1988; Kimaro et al. 2005; Muwanga, Schuman & Biryabarema 2001; Ngecu & Mathu 1999; Omara-Ojungu 1992; Temple & Rapp 1972), and this is largely attributed to high annual rainfall, steep slopes, land use change, high weathering rates and slope materials with a low shear resistance or high clay content (Knapen et al. 2005). Vegetation cover is an important factor influencing the occurrence and movement of rainfall triggered landslides, and changes to vegetation cover often result in modified landslide behaviour (Glade 2002). The change in land use has been recognised throughout the world as one of the most important factors influencing the occurrence of rainfall triggered landslides. Land-use change (conversion of forest into agricultural land), especially on the foot slope, causes disturbance to the natural balance of the slope (Paudel, Joshi & Devkota 2006). In Uganda, the most vulnerable area is Mt Elgon and, in particular, Bushika Subcounty in Bududa district. The narrations by local people indicate that landslides are an old problem in this area and have caused destruction of much property and loss or injury to human life (Kitutu 2006). More than 500 people have lost their lives in the last half a century. The exceptional heavy rains of 1997–1999 moved a large amount of slope material downslope. In 1997, at least 48 people were killed, the crops and dwellings of 885 families disappeared from the map, 5600 people became homeless, arable land was reduced causing land scarcity and property conflicts, water supplies were polluted with a consecutively epidemic and, finally, the districts were hit by food shortage (Knapen et al. 2006). The landslides of 2010 in Namesti caused tremendous havoc: moved massive fertile soils downslope, destroyed farmlands, killed over 300 people and led to displacement (Atuyambe et al. 2011; Mugagga, Kakembo & Buyinza 2012).

The most recent wave of landslides in the country particularly in the late 1990s and early 2000s is mainly attributed to multiple factors such as climatic changes (e.g. El Nino rains) (Ngecu, Nyamai & Erima 2004), deforestation, deeply weathered soils underlain by tertiary and Pleistocene volcanic rocks, steep topography and human activities such as cultivation (Kitutu et al. 2009; Knapen et al. 2005; Mugagga 2011; Nakileza 2007). Human activities, such as poor logging practices and overplanting on steep slopes, accelerate landslide occurrence (Chang & Slaymaker 2002). Studies in Bududa district by Claessens et al. (2007) using the landscape process modelling at multi-dimensions and scales (LAPSUS-LS) model found that, in general, shallow landslides occur at a relatively large distance from the water divide, on the transition between steep concave and gentler convex slope positions. This trend points to concentration of (sub) surface flow as the main landslide controlling mechanism. This is in line with Highland and Bobrowsky (2008) who noted that slope saturation by water is a primary cause of landslides.

Considering the scarcity of land for cultivation and settlement, recovery and re-use of the slide scars is very important. Further, the Hyogo Framework of Action 2005–2015 and the Sendai Framework for Disaster Risk Reduction 2015–2030 emphasise the importance of improved resilience at national and local community level. The concept of resilience is variously defined but covers the capacity of public, private and civic sectors to withstand disruption, absorb disturbance, act effectively in a crisis, adapt to changing conditions, including climate change, and grow over time (Martin-Breen & Anderies 2011). In this study, enhancing resilience was considered as the capacity of a community to organise itself to reduce the impact of landslide disasters by protecting lives, livelihoods, homes, assets, basic services and infrastructure. Capacities include skills, knowledge, resources, practices and networks. Landslide scar rehabilitation is part of the effort to reduce further risks and live with the changing conditions. However, efforts to rehabilitate the scars have been hampered by inadequate resources and information, which is partly explained by limited research. There is particularly dearth of information on management of landslide scars. The article aims at (1) estimating the volume of soil displaced by landslide in selected area in Bududa district on Mt Elgon in Uganda, (2) assess the perceived damage caused by the landslides, (3) identify the method of use by the local communities in their rehabilitation and (4) assess their degree of rehabilitation.

## Methodology

### Description of the study area

**FIGURE 1:**
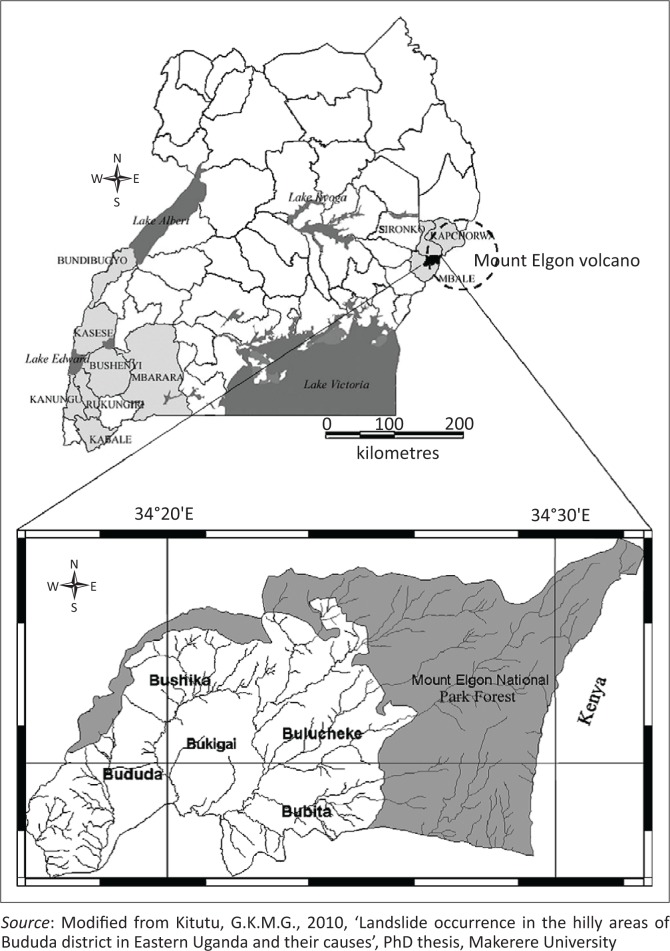
Location of Bushika in Mt Elgon, Uganda. Bududa district on Mt Elgon in Uganda and Bushika Subcountry in mid to upper catchment of River Manafwa.

The study was undertaken in Bushika Subcounty, Manafwa district in eastern Uganda. The area is located between 1° 03′ 1° 05′ N and 34° 19′–34° 22′ E (Figure 1). Rivers and streams from Mt Elgon densely dissect it, thus the rugged topography characterised by steep slopes. The steep slopes in the north of the area grade into gentle slopes and broad valley in the south just bordering the carbonite ring. More than 60% of the land is located on slopes > 15°, which is critical for accelerated mass movement. The area is an inherently unstable region, where human interference plays a major role. Steep, plan concave slope segments at a certain distance from the water divide and oriented to the dominant rainfall direction (north to northeast) are the most sensitive to mass movement throughout the study area (Knapen et al. 2006). Other recent studies (e.g. Mugagga 2011) are in agreement with this observation. The main lithology is the fenitised basement rocks, which are the oldest and of Archean age. Landslides are reported to be common in this zone (Kitutu 2006). The soils, according to Ollier and Harrop (1959), belong mainly to Bududa and Bubutu series, which consist, respectively, of clay loam originating from Elgon volcanic or the basement complex and of the non-laterised brown sandy clay loams originating from the basement complex. The major soil types are cambisols, lixisols, ferralsols, leptosols, gleysols, nitisols and acrisols and are mainly conditioned by topography and wet tropical climate (Kitutu et al. 2009). The main land use type is agriculture (farming and small-scale grazing) and forest (national park). The dominant crops are perennial (banana and coffee), which are intercropped with beans and maize. The area is among the most densely populated region of Uganda. The Uganda National Bureau of Statistics (UNBOS 2002) estimated the average density of population at 952 people/km^2^, but in some parishes, it stood at 1300 people/km^2^. The UNBOS 2014 census data revealed high population density in these areas. This high population density exerts pressure on the land; land scarcity is acute and a number of people settled or cultivate on steep slopes greater than 60%. Population pressure and its induced land scarcity are among the factors preventing people from abandoning the most landslide-prone areas (Knapen et al. 2005; Mugagga et al. 2012). Highland and Bobrowsky (2008) reported that populations expanding onto new land and creating neighbourhoods, towns and cities are the primary means by which humans contribute to the occurrence of landslides through disturbing or changing drainage patterns, destabilising slopes and removing vegetation.

### Estimation of the volume of soil displaced by landslide events

A field survey approach was adopted to quantify the volume of soil displaced by landslide. All the scars on agricultural lands in Bushika Subcounty were identified with the help of local farmers and members of Shunya Yettana community-based organisation. Classification of the landslides was based on Varnes (1984), and Temple and Rapp (1972) proposed the approach. The dimensions (width, length and depth) of scars were determined using a tape measure, a clinometer was used for slope gradient determination and a GPS for geo-referencing the different studied scars. The landscape, geomorphological features, soil and land use were also characterised.

### Identification and performance of the rehabilitation methods used by local communities

Landslide scars undergoing rehabilitation through individual or communal efforts were also surveyed in other areas in Bududa and Manafwa districts. Field observations, focus group discussions and key informant interviews with the district officials were used to identify the methods used by the community to rehabilitate the scars. During field observation, the focus was on the type of plant species used by the communities and the type of soil erosion processes occurring on the rehabilitated land. Group discussions and informal interviews with the local community in Bushika and other areas were used to capture their perception on the performance of management practices applied on landslide scars and related challenges faced. A total of 30 farmers were purposively sampled and informally interviewed about their knowledge of landslide risks, initiative and land use practices on scars and constraints to landslide scar management. Secondary data sources included grey literature and published materials in peer reviewed journals.

Composite soil samples were collected on selected best representative rehabilitated landslide scar in Bushika; on the upper, mid and lower sections to obtain insights into changes in soil properties. Soils sampled were analysed for pH, organic matter, texture and a few chemical properties based on standard procedure (Okalebo, Gathua & Woomer 1993).

## Results and discussion

### Quantification of volume of soil moved through landslide in selected landslide scars in the subcounties of Bududa district

Table 1 shows the characteristics of the surveyed landslide scars in Bududa district. The major types of landslides identified were bottle slides, mudslides and shallow slides. The sheet slides were rather shallow (< 1.5 m), occurred on mid slopes and mainly removed the soils and shallow-rooted crops such as maize, beans and bananas.

**TABLE 1:** Slide type, dimensions and land use types in different villages in Bududa district.

Slide code	Date of occurrence	Type of slide	Village	GPS location (UTM)		Altimeter (m)	Slope (%)	Length (m)	Width (m)	Depth (m)	Land use
				x	y						
SL1	1998	bsl+ms	Bunakasala	653 037	117 801	1628	70	175	19	12	b+c
SL2	2001	bsl+ss	Bunambazu1	652 567	118 038	1709	50	50	30	2	b+c
SL3	1998	bsl+ms	Bubayela	652 733	118 015	1757	60	-	-	1–2	b+c
SL4	2001	bsl+ms	Bunambazu2	652 454	118 269	1737	60	80	40	15	b+c
SL5	1970s	bsl+ms	Bunambazu3	652 684	118 400	1814	70	150	120	5	b+c
SL6	-	bsl+ms	Bunambazu4	652 724	118 685	1910	70	80	8	3	b+c
SL7	1997	bsl	Bunabutiti	648 901	117 672	1427		500	80	5	b+c+m
SL8	1997	bsl+ms	Buriri	647 207	118 653	1655	80	90	40	10	b+c
SL9	1997	bsl+ms	Buriri	647 218	118 574	1664	80	60	50	5	b+c
SL10	1997	bsl+ms	Bumushisho	647 297	116 130	1488	55	110	30	3	b+c
SL11	2011	ms	Namesti	658 424	113 700	-	70	>1000	300	3–5	m+ca+IP+be+O
SL12	2015	-	Bushika and Bunanyuma	647 446	116 708	-	25	106	40	-	c+cs+b
SL13	1997	-	Bukalasi and Reyeri	655 022	112 219	1634	46	55	50	7.5	c+cs+m+be+E
SL14	1997	-	Bumayoka and Bukhadye	654 620	113 936	-	55	71	185	1.5	m+ca+E
SL15	1998	-	Bumayoka and Nangobe	655 648	114 934	1796	45	44	175	7	m+be+cs
SL16	2013	-	Bumayoka and Bukayenjele	654 998	114 528	1693	40	40	66	2.3	m+E
SL17	1997	-	Weswa and Makhonje	649 235	108 270	1450	51	78	190	5.3	cs+b+be
SL18	1999	-	Buwali and Buwashi	653 753	111 491	1454	43	62	226	4	m+gn+p+c+E
SL19	1997	-	Bumulika	654 456	115 320	1627	47	50	76	3.5	m+cs+b
SL20	1997 and 2016	-	Naboshi	649 193	117 456	-	10	86	332	6.3	Abandoned

Note: The GPS, the Geographical Position System, refers to the geographical location of the landslide scar.

b, banana; be, beans; bsl, bottle landslides; c, coffee; Ca, cabbages; Cs, Cassava,; E, Eucalyptus; gn, Ground nuts; IP, Irish potatoes; m, Maize; ms, mud landslides; O, onions; p, Peas; ss, sheet landslides.

The bottle slides were deep scouring (> 5 m – 15 m) and mainly occurred on upper slopes. Some of these slides changed to mudflows (bsl + ms) on the lower sections in the valleys or drainage channels because of the increase in water content in the soil mass. Most of the slides (> 90%) belong to the category of the ‘bottle slides’ (bsl, ms) (Figure 3) and ‘sheet slides’ (ss) (Table 1) according to the literature.

**FIGURE 2:**
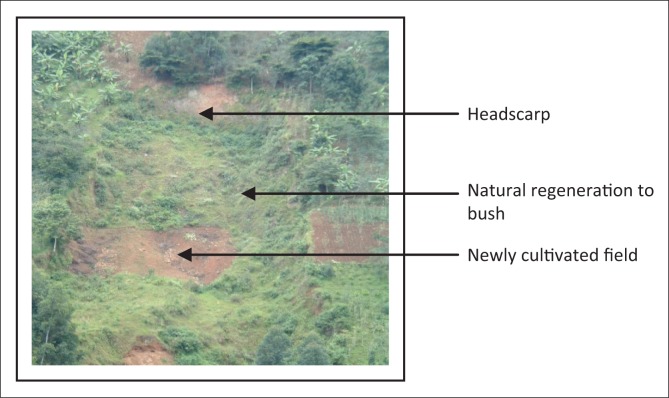
Bottle-type landslide in Bushika; 9 years after its occurrence.

**FIGURE 3:**
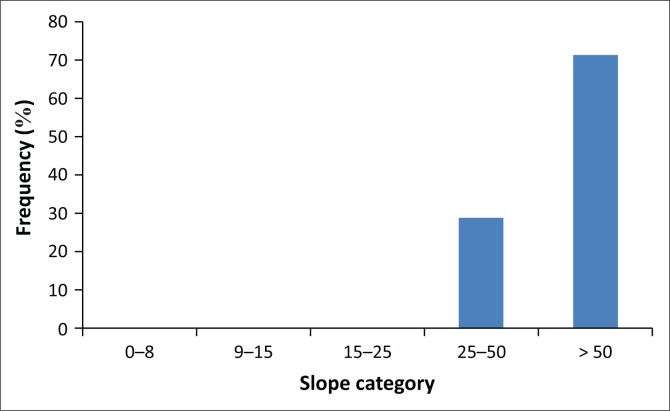
Landslide occurrence on different slopes of Bushika Subcounty.

**FIGURE 4:**
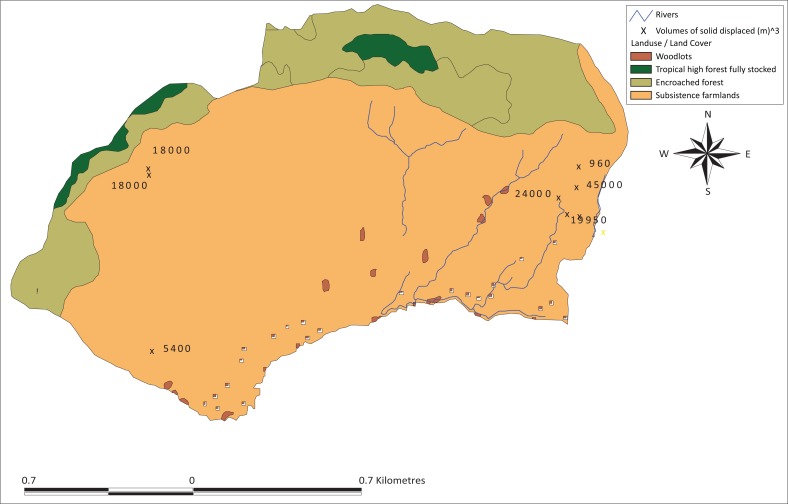
Volume (m^3^) of materials lost through landslide under different land use or cover in Bushika Subcounty.

The majority of the scars occurred in 1997–1998, largely because of the effect of the El Nino rains, as also argued by Ngecu et al. (2004). These slides have tended to occur in the neighbourhood of the past landslides (Figure 2) on slopes of greater than 50% (for 70% of the cases observed in the field) (Figure 3). This could be explained by reduced shear stress and slope strength because of the human disturbance and slope steepness. The volume of materials, which was lost for the surveyed scars, ranged from 960 m**^3^** to 45 000 m**^3^** (Figure 3). Nearly 100% of the observed landslides occurred outside the forested areas on agricultural land dominated by banana intercropped with coffee (Figure 4).

**FIGURE 5:**
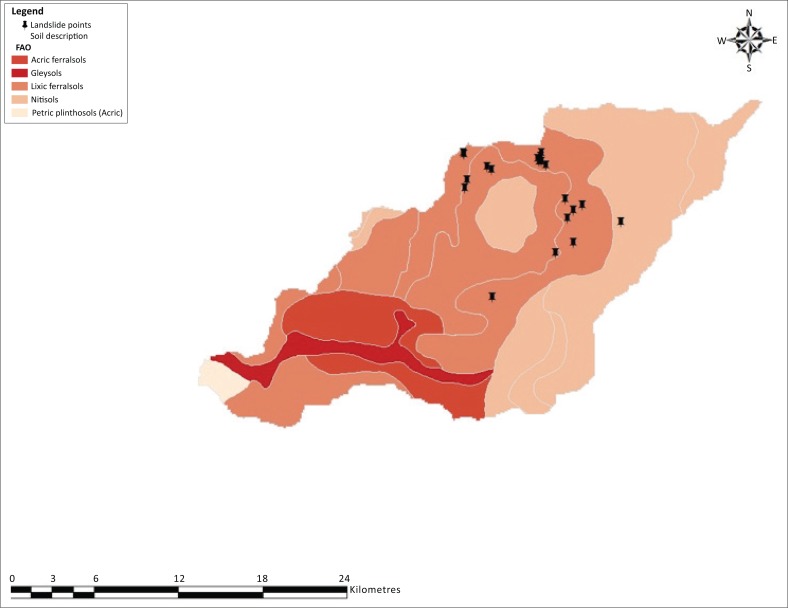
Distribution of landslide scars on different soil types in the Manafwa catchment study area.

Figure 5 shows the distribution of landslides on different soils in mid-part of Manafwa catchment in Bududa and Manafa districts. There were more slides on Haplic type of soils (red–yellow clay loams) compared with other soil types. The Haplic soil-type area also coincides with steep slopes. Kitutu et al. (2009) found that the soil type had no influence on landslides, but soil texture seems to be of significance in this zone. Studies in the southern part of the area by Mugagga et al. (2012) provide more insights into the influence of soil properties on landslides. He observed that the soils had high plasticity, water retention and low permeability. Landslides in the western zone of Bududa and, therefore, Bushika are because of soil horizon stratification that favours water stagnation in the lower horizon. That is, landslides are majorly confined to places where there is water stagnation or hydrological saturation in the lower soil horizons. Our observation of scars in this area noted the presence of streams flowing over weathered rock plane. We noted that the destruction of plant cover could have reduced the stability of soil particles and contributed to increased infiltration, hence high subterranean flows that induced slides.

### Damage caused by landslides in the study area

Landslides are among the most frequent and damaging environmental hazards in the study area, causing loss of life, loss of livelihood and disruption to road traffic and economic activity. The occurrence of landslides is thus associated with environmental, socio-economic and political problems, both directly and indirectly. Table 2 summarises the problems identified during participatory rural appraisal (PRA) discussions and through informal interviews. The problems include loss of human life, displacement of people, destruction of property, accelerated geomorphic processes and reduced water quality through increased siltation load.

**TABLE 2:** Response on the main landslide-related problems experienced in Bududa.

Problem	Extent and nature of the problem
Destruction of human life	Serious, causing traumatising effect in children and adults who lost their relatives
Displacement of people	Many have been forced to vacate their land or abandoned it
Destruction of property	Mainly houses, crops, bridges, soil
Accelerated geomorphic processes	Sheet and rill erosion mainly on bare scars
Sedimentation	Loading of rivers with sediments from scars

Landslides displace people; thus, increased pressure is shifted to other cultivated lands, which coupled with poor conservation methods leads to soil fertility decline. Poor soil fertility was ranked as a major constraint to agricultural production. Studies conducted in the area by Bamutaze (2010) revealed serious soil and nutrient loss, particularly from annual crop farmlands. Loss of human life and property causes socio-psychological problems; most of the victims were reportedly traumatised because of loss of their relatives and property. This was reported to have negatively affected their level of investment in the land.

Displacement of farmlands downslope commonly results into conflicts. Conflicts reportedly often arise between land users on the lower and higher slopes, whenever farmlands from upslope slide and displace or burry those on the low-lying areas. Common cases of that kind reported to local councils (LC 1) concern making an appropriate decision on identifying the right owner of the land above the buried one. This remains a tricky issue.

Landslides lead to loss of fertile soils upslope; the soils are detached and moved downslope or even lost when transported to streams and rivers. This exposes infertile soils and sometimes bedrock at the onsite positions. Infertile soils including boulders are deposited downslope covering fertile soils and vegetation. However, in other situation, fertile soils are deposited depending on the nature of the debris.

Quantification of the resulting damage, however, has not been undertaken on a detailed scale. District officials have only attempted to perform rapid assessments to provide data for emergency response. Even when detailed assessments are undertaken using such tools as total economic valuation, it is hard to quantify all aspects related to environmental phenomena such as landscape value, biodiversity loss and ecosystem services.

### Land management practices for enhancing resilience on the landslide scars

**FIGURE 6:**
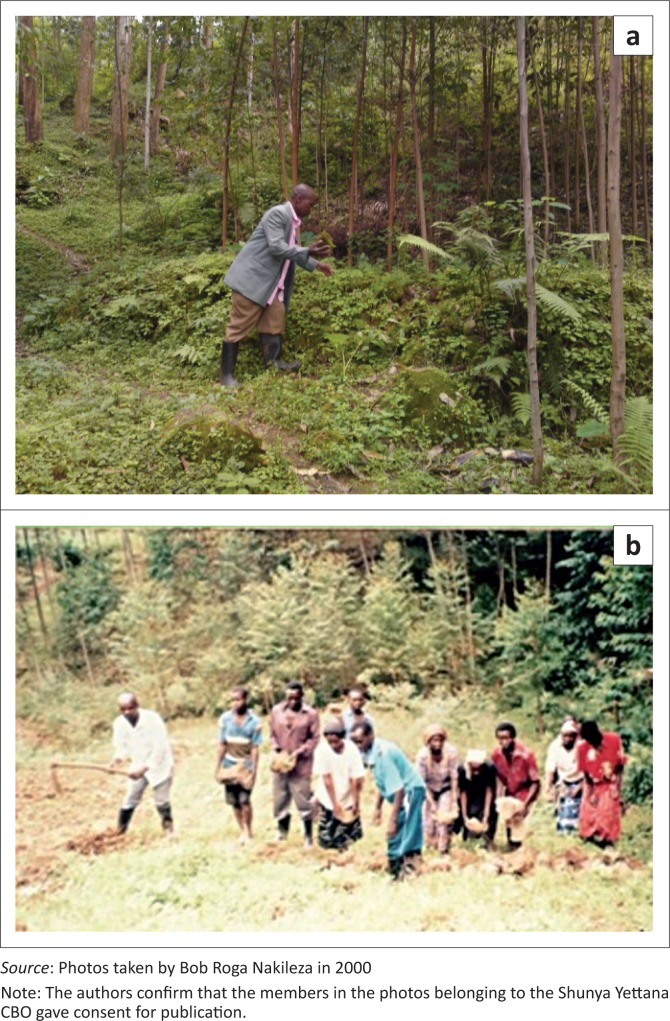
(a) Stone terrace (where the person is pointing) and eucalyptus trees planted to stabilise the soils on a scar in Bushika and Bunakasala villages. Note also the underground dense herbaceous growth, which is important for enhanced stability of the soil. (b) The local community members of Shunya Yettana working as a team in digging, collecting and assembling stones along a contour on the landslide scar.

Interviews and discussions revealed that 50% of the participants have landslide scars on one or more of their fragmented plots. Most of the people have plots in different locations including steep slopes, hence increased chances of experiencing landslides. The majority of the old landslide scars (60%) are either abandoned or cultivated with annual crops (e.g. maize and beans) (Table 1). Nevertheless, farmers reported increased and improved scientific understanding of landslide process and risk reduction following workshops held during the implementation of the United Nations Development Programme/Small Grants Programme (UNDP/SGP) project and efforts by the National Environmental Management Authority and Bududa District Environmental Office. In the UNDP/SGP project implementation, farmers in Bushika Subcounty were exposed to the concept of watershed management, which was vital in bringing different stakeholders together in assessment of the environmental risks, problems and planning. Improved knowledge of landslides, rehabilitation of scars and risk reduction are relevant parts of the local community capacity building for increased resilience to landslide impacts. As well, economic benefit through carefully planned harvest of planted trees on landslide scars or landslide-prone areas by farmers can provide returns that motivate farmers for further investment in rehabilitation of landslide-affected lands. This is consistent with studies by Twaha et al. (2012) who established that farmers in Mt Elgon were largely interested in planting trees for economic gains. In general, most of the farmers have so far employed the following management practices to stabilise the landslide scars:

- Stone bunds – Under technical advice, stone bunds were adopted by the local community on one landslide scar in Bushika to stabilise the slope hence also control erosion (Figure 6). This was perceived to have greatly controlled erosion, thus permitting revegetation growth. Stone structures have been applied elsewhere and proved to work. For instance, WOO (1992) in Korea adopted Stone soil arresting structures for rehabilitation of the southern hillslope scars. Young *Pinus densiflora* and *Pinus koraiensis* trees (tree height 1.5 m – 2.0 m, 10–14 years of age, 1.5 m – 1.8 m distance in a row) were planted on each step of the structures. He strongly recommended rehabilitation of landslide torrents using reinforced concrete structures and rock fall retarding screens.
- Agroforestry – Planting of trees such as eucalyptus (Figure 6) has been taken up by farmers on a number of scars because of fast growth rate and anticipated economic benefits. A few of the respondents who have planted eucalyptus explained that the roots penetrate deep into the soil and are therefore suitable for stabilising the soil. However, growing of eucalyptus trees has also raised questions concerning its rapid water uptake causing deficiencies for other uses. This calls for further investigations to inform decision makers. Indigenous tree species such as *Cordia africana* are recognised as useful, but they are slow in growth. *Albizia julibrissin* was reportedly being encouraged in coffee and banana-cropping system, though had not been planted on the scars. It provides shade to the coffee beside leaf litter for enriching soil organic manure. Though not measured in this study because of limited resources, literature reveals that tree roots enhance soil shear strength when the root network penetrates a potential failure surface. High tensile root force contributes to a potential slide mass that should increase with increasing area of root intersection (Roering et al. 2003). Trees contribute to landslide rehabilitation as also noted by Kervyn et al. (2015) but have some limitations. In a policy brief by Food and Agricultural Organisation (FAO), it is noted that trees reduce landslide risks by lowering the soil moisture levels through interception, evaporation and transpiration mechanism. However, it was further noted that trees could also increase landslide risks by imposing overload on unstable slopes and via wind-related effects. Trees are, therefore, unlikely to prevent or minimise deep landslides on very steep slopes. Further research focussed on this should be of great significance. On slopes undergoing restoration, management of hydrological processes is also fundamental for the success of a soil bioengineering structure and vegetation establishment (Stokes et al. 2014).
- Napier grass (*Pennisetum purpureum*) has been planted though on a very limited scale on shallow scars. It is largely used as fodder and for stabilising the soil through the dense root network. Napier grass is a tall, perennial grass indigenous to tropical Africa that performs well to an altitude of 2000 m or beyond. It has the advantage of withstanding repeated cutting, and four to six cuts in a year can produce 50–150 tonnes fresh herbage per hectare (Purseglove 1972). However, repeated cutting and removal of Napier grass can lead to nutrient depletion. This was one of the problems farmers in the study area reported when they plant it on their plots. Mwendia et al. (2007) observed that recycling cattle manure on Napier grass should largely replenish phosphorus and potassium removed by cut and carry systems.
- Natural plant regeneration – As a traditional practice, the landslide scars are commonly abandoned for some time, ranging from as short as 2 years up to a decade or beyond depending on many factors (e.g. land scarcity, depth of the scar) to allow for recovery and to avoid further problems of sliding (secondary slides). This provides opportunity for natural plant regeneration or restoration of the degraded areas. Planting or encouraging natural growth of vegetation can be an effective means of slope stabilisation (Highland & Bobrowsky 2008). As indicated by Temple and Rapp (1972), colonisation of slide scars by vegetation and the development of soil on their surfaces will occur at variable rates, depending on a wide range of factors such as altitude, depth of the slide scar in relation to soil and/or regolith and the availability of soil moisture and of colonising species. This is further supported by Gecy and Wilson 1990 cited in Stoke et al. (2014) who argue that revegetation patterns will depend largely on the response of both vegetative re-sprouts and seedlings, the number of disturbances already incurred at the site, the initial species composition before the debris flow and the position of the regrowth along the debris flow. Some of the plant species that had colonised the scars surveyed in the study area are listed in Table 3. Preliminary observations revealed that the plants in the neighbouring farmlands or bushland outside the scars have enhanced colonisation of scars. There is, however, need to accelerate the restoration through selective planting of trees and shrubs that can provide multiple benefits to farmers. This is supported by McBride and Voss (1990) who observed that planting of agroforestry species useful for fuel wood and/or forage is most likely to be adopted by farmers as a low-cost but expeditious strategy for landslide rehabilitation. They further argue that manipulation of temporal patterns of natural plant establishment is directly relevant to ecosystem restoration, a logical focus being the immediate re-establishment of woody vegetation after disturbance on very steep slopes.

**TABLE 3:** Some of the common species identified on landslide scars and their uses.

Species name	Common or local name	Uses
*Stipa nervosa* var. *nervosa*	Lubembe	Thatching huts
*Vernonia amygdalina*	Mululuza	Treating malaria
*Sesbania sesban*	Manafwa yaleta	Stakes, fuelwood
*Cyperus* spp	Sedges	Mulching, thatching huts
*Impatiens* sp.	-	-
*Maesa lanceolata*	-	-
*Paullinia pinnata*	-	-
*Eucalyptus mynaceae*	Kalintusi	Poles, firewood, medicinal
Pteridophytes	Luzanzasi	Sweeping, wrapping things
*Cordia africana*	Khuyiyi	Fuel wood
*Asteraceae conyza sumatrensis*	*-*	-
*Digitaria abyssinica, Digitaria scalarum*	Couch grass or Lumbugu	Grazing

### Soil characteristics of landslide scars

**FIGURE 7:**
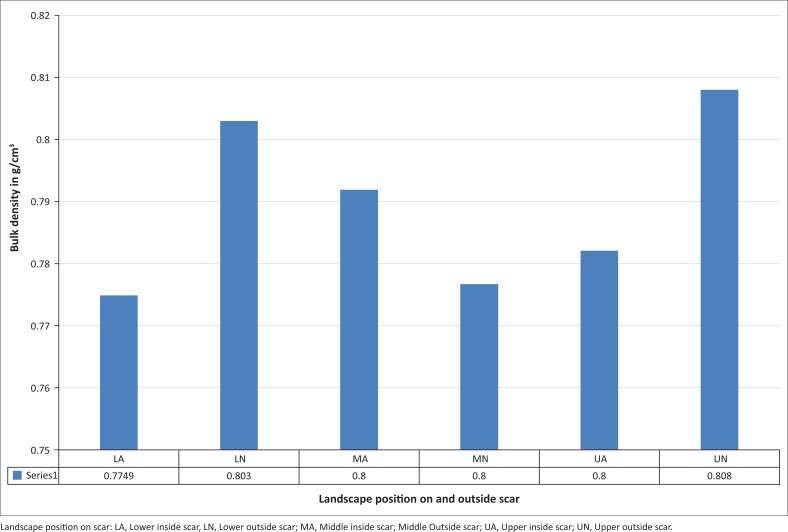
Bulk density of the top soil layer in the landslide-prone area of Mt Elgon.

A summary on the soil characteristics of landslide scars and the areas in the neighbourhood not affected by landslides is provided in Table 4. Generally, areas affected by landslides have a lower clay B-horizon, except at the lower slope, where the clay pan was sandwiched between two clay-loam textured B-horizons. The areas that were not affected by landslides were in general uniformly textured, except at the upper slope, where a clay pan was observed immediately after the topsoil layer. Increased water content mobilises the clay as it is absorbed into the clay structure. Thus, clay-rich materials have a high potential for accelerated deformation and ultimate failure in the presence of excess water. Generally, the organic matter levels are very high across the soil profile. However, clay layer in the affected area tended to have relatively low organic matter compared with the layer above or below it, whereas the non-affected area tended to have a quasi-uniform level of organic matter content. Figure 7 depicts the bulk density of the topsoil layer for the different landscape positions. Generally, the topsoil layer is very porous. The implication of this is that in the non-landslide-affected areas the flow of water is quasi-uniform across the soil profile, whereas in the landslide-affected areas water pressure builds up on top of clay layer because of its lower permeability. This is in line with observation made by Kitutu (2010) and Mugagga et al. (2012) in the same region. Bizimana and Sonmez (2015) also noted that high plasticity clay soils contribute to landslides. Apart from the influence of soils, topographic and geologic features as controlling factors for slope instability, the most common landslide triggers are intense rainfall events (e.g. Bizimana & Sonmez 2015; Crosta 1998). Numerous researchers have shown the relationship between landslide occurrence and intense rainfall periods (e.g. Azzoni et al. 1992; Canuti, Focardi & Garzonio 1985; Ferrer & Ayala-Carcedo 1997; Finlay, Fell & Maguire 1997; Hoseop, Wonseok & Enukwa 2016; Hosseini & Hosseini 2011; Kitutu 2010; Polemio & Sdao 1999; Zezere, Ferreira & Rodrigues 1999). Although steep topography has classically been viewed as the main indicator of susceptibility to rockfall and deep-seated landslides, intense rainfalls produced during wet seasons can be the triggering factor in the initiation of slope failures. Rainfall events can also produce an increase in the rate of movement on landslides.

**TABLE 4:** Soil properties on different slope positions of the landslide scar and adjacent area in Bushika.

Landscape position	pH	OM (%)	*N*	*P* (ppm)	Ca	Mg	*K*	Sand (%)	Clay	Silt	Textural class
Upper slope	5.6	4.1	0.22	22.7	2639.0	780.0	280.5	39.7	34.3	26.0	Clay loam
	5.4	3.0	0.16	8.0	2150.0	695.0	270.0	39.7	34.3	26.0	Clay loam
	5.3	2.9	0.17	7.9	2273.8	803.0	216.44	29.7	44.3	26.0	Clay
Outside scar upper slope	5.5	5.3	0.25	18.3	2750.0	880.9	217.0	29.7	36.3	34.0	Clay loam
	5.8	5.2	0.26	1.7	2986.1	870.0	285.7	29.7	42.3	28.0	Clay
	5.7	5.3	0.26	2.1	2840.0	889.1	264.0	39.7	30.3	30.0	Clay loam
	5.5	5.7	0.27	8.8	2883.6	852.0	239.0	41.7	28.3	30.0	Clay loam
Mid slope	5.8	5.7	0.27	5.1	2772.5	800	292.55	37.7	34.3	28.0	Clay loam
	5.9	4.1	0.21	19.4	2751.8	771	285.9	45.7	30.3	24.0	Sandy clay loam
	5.8	3.8	0.21	16.4	2551.8	744.0	292.7	39.7	40.3	20.0	Clay
Outside scar mid slope	5.8	3.9	0.20	4.7	2595.9	897.0	248.3	35.7	34.3	30.0	Clay loam
	5.5	3.5	0.18	42.0	2193.6	680.0	220.0	29.7	36.3	34.0	Clay loam
	5.2	4.4	0.22	29.4	2370.0	637.0	245.4	39.7	32.3	28.0	Clay loam
	5.0	4.7	0.24	14.2	2670.0	713.0	212.7	39.7	32.3	28.0	Clay loam
Lower slope	5.0	3.0	0.18	32.9	1784.8	535.6	248.0	41.7	36.3	22.0	Clay loam
	5.2	1.4	0.12	22.9	1430.0	469.6	235.0	39.7	40.3	20.0	Clay
	6.3	4.6	0.22	27.0	3010.0	906.0	295.0	39.7	36.3	24.0	Clay loam
Outside scar lower slope	5.8	4.2	0.23	33.0	2120.0	698.3	233.6	29.7	42.3	28.0	Clay
	6.0	3.8	0.21	23.9	2503.2	830.0	300.8	29.7	36.3	34.0	Clay
	5.8	4.9	0.24	23.8	2840.0	860.0	269.7	19.7	50.3	30.0	Clay
	6.0	4.3	0.21	4.4	2821.8	906.0	303.0	39.7	52.3	8.0	Clay

### Challenges to the management of landslide scars

Based on discussions with the local communities and authorities, numerous challenges faced in the restoration of degraded landslide scars were identified as outlined below:

- Occurrence of secondary slides remains a hindrance particularly on steep slopes and where there are deep head of the slides. Deep head of the slides does not easily permit the use of methods such as excavation for stability. Many of the affected farmers have hesitated to embark on the rehabilitation of the scars for fear of further destruction of crops, trees or even triggering more slides.
- Lack of funds to secure the necessary expertise and materials (e.g. cement, pipes, wire mesh) for installation of stabilising structures or embankments such as gabions. Gabions are wire mesh boxes filled with cobble-sized rock (10 cm – 20 cm in size) (Chatwin et al. 1994). Gabions can be easy and flexible to use where cobbles abound, but the mesh may be more expensive. They have been, however, successfully used in the area of Bukavu in the Democratic Republic of Congo (Ischebeck, Kabazimya & Vilimumbalo 1984). External support from non-governmental organisation and other development partners would be required if such a practice was to be widely adopted by farmers in this study area.
- There is also lack of clearly known or established best approaches for the local communities to learn from and adopt. This partly arises from lack of investment in research testing the best landslide scar management practices in this area. The limited experimental soil movement research in Mt Elgon (e.g. Bamutaze 2010; Nakileza 1992; Semalulu et al. 2015; Tenywa 1993) has been more academic, short-lived and focussed on soil erosion outside the slide scars.
- There is limited existing local community strategy to mitigate the risks of landslides and rehabilitation of the scars. Individual and isolated interventions (e.g. by Shunya Yettana) have emphasised planting of eucalyptus trees. This demonstrated good practice though on a small scale. There is a challenge considering that landslide effects are spread over catchments in both low and high lands affecting a wide range of people either directly or indirectly. Thus, a well-coordinated community strategy based on sound information and knowledge of landslides is therefore desirable and needs to be pursued.
- Land scarcity is a serious problem. Scarcity of land has compelled some farmers to encroach on fragile steep slopes for cultivation. Land on steep slopes is very sensitive and can easily induce further landslide incidences. This is also in line with the argument advanced by Knapen et al. (2006). Thus, care should be exercised when using farmlands on steep slopes to ensure minimal disturbance. Alternatively, use of sustainable land use practices such as minimum tillage can avoid accelerated degradation.
- Persistent improper land management practices remain a problem. Practices such as over-cultivation, digging upslope, uncontrolled cutting down of trees on farmlands located on steep slopes and lacking surface run-off control are common in the mountain area.
- Ephemeral cracks on slopes present a challenge in that they are initiated below the surface and therefore rarely seen until when in advanced stages. The presence of cracks depicts soil movement below the surface and presents easy points for water percolation.

## Conclusion

Landslides cause serious problems, which undermine the productive capacity of the land and hence contribute to the poverty experienced in many areas on Mt Elgon slopes. This study set out to characterise the landslides, establish the perceived damage and community efforts in stabilising the landslide scars plus challenges faced in Bududa district. Field surveys, observations and discussions were applied in gathering the relevant data.

The results indicate the dominance of landslide scars on steep slopes ranging from 50% to over 80% and covered mainly by ferralsols and nitisols. Fewer landslides were identified in the forested areas pointing to the effect of tree root stability among other factors as also noted by Mugagga et al. (2012). Most landslide scars were in the cultivated areas grown with bananas, maize and Arabic coffee, hence indicating the significance of anthropogenic influence. Based on the reviewed literature, the main factors triggering landslide occurrences in the area are heavy rainfall and disturbed steep slopes.

Knowledge and understanding of landslide controlling factors is very important in drawing up strategies for mitigation and restoration of the scars. The most seriously perceived damage by landslides was the loss of human life and household property. Thus, designing and/or implementing safeguard measures against such losses are very important.

Apparently, there is limited on-going effort to address landslide and related degradation challenges. The initiatives such as stone terraces and enriched tree planting adopted by the local community particularly in Bushika have demonstrated that the scars can be healed faster for productive use. Such initiatives combined with awareness and participatory work by communities increase their preparedness to landslide disaster risk in the area. Restoration of degraded landslide scars is vital, considering the high population pressure in the area and the need to achieve improved environmental stability and livelihood. However, it is important to consider the various environmental and socio-economic challenges to mitigate the landslide problems in such areas. Emphasis on affordable low-cost technologies (e.g. planting trees and use of stone terraces) for small-scale farmers in the rehabilitation of landslide scars is a favourable approach. Further research is needed to investigate the restoration and regeneration rates in the scars in various socio-economic and environmental settings in the mountain regions.
