# Corrigendum: Enhancing resilience to landslide disaster risks through rehabilitation of slide scars by local communities in Mt Elgon, Uganda

**DOI:** 10.4102/jamba.v9i1.568

**Published:** 2017-11-30

**Authors:** Bob R. Nakileza, Mwanjalolo J. Majaliwa, Abu-Baker S. Wandera, Clare M. Nantumbwe

**Affiliations:** 1 Department of Environment Management, Makerere University, Uganda.; 2 Department of Geography, Geo Informatics and Climatic Sciences, Makerere University, Uganda.; 3 The Global Environment Facility Small Grants Programme/United Nations Development Programme, Uganda.

In the version of this article initially published, Mwanjalolo J. Majaliwa’s first name was misspelled as ‘Mwajalolo’ and Abu-Baker S. Wandera’s first name was misspelled as ‘Abu’. The third affiliation was changed and updated to ‘The Global Environment Facility Small Grants Programme/United Nations Development Programme, Uganda’. The errors have been corrected in the PDF version of the article. The authors apologise for any inconvenience that this omission may have caused.

